# Acid-Suppressive Therapy Choice and Risk of Treatment Escalation in Inflammatory Bowel Disease: A Real-World Comparative Retrospective Study to Inform Personalized Treatment

**DOI:** 10.3390/jpm16040193

**Published:** 2026-04-01

**Authors:** Yan Sun, Donovan Veccia, Gengqing Song, Nisheet Waghray

**Affiliations:** 1 Department of Internal Medicine, MetroHealth Medical Center, Case Western Reserve University, Cleveland, OH 44109, USA; sunjxz@yahoo.com; 2 Department of Gastroenterology and Hepatology, MetroHealth Medical Center, Case Western Reserve University, Cleveland, OH 44109, USA; dveccia@metrohealth.org

**Keywords:** inflammatory bowel disease, proton pump inhibitors, histamine-2 receptor antagonists, treatment escalation, *Clostridioides difficile*

## Abstract

**Background/Objectives:** Proton pump inhibitors (PPIs) are known to alter gut microbiota composition; however, their association with disease courses and outcomes in patients with inflammatory bowel disease (IBD) remains uncertain. Our aims were to evaluate the association between PPI use and treatment escalation, *Clostridioides difficile* infection, and healthcare utilization in IBD. **Methods:** We conducted a retrospective cohort study on the TriNetX platform. IBD patients with PPIs or histamine-2 receptor antagonists (H2RAs) were matched one-to-one using propensity scores. Outcomes included initiation of corticosteroids, biologic therapy, *Clostridioides difficile* (*C. difficile*) infection, and healthcare utilization. Outcomes were assessed during the 0–12-month and 3–12-month follow-up windows. Associations were estimated using odds ratios (ORs) and hazard ratios (HRs) with 95% confidence intervals. **Results:** After matching, 12,808 patients were included in each group. During 0–12 months of follow-up, PPI use was associated with higher odds of systemic corticosteroid exposure (OR 1.56, 1.35–1.79), biologic therapy initiation (OR 1.99, 1.72–2.29), C difficile infection (OR 1.42, 1.18–1.70), and healthcare utilization (OR 1.18, 1.03–1.36) compared with H2RA use. Time-to-event analyses showed persistent associations with systemic corticosteroid exposure (HR 1.50, 1.31–1.72) and biologic therapy initiation (HR 1.91, 1.66–2.19), with attenuation of associations for infection and healthcare utilization in 3–12-month lag-time analyses. Similar patterns were observed in ulcerative colitis and Crohn’s disease subgroups. **Conclusions:** PPI was associated with higher risks of treatment escalation and *C. difficile* compared with H2RA in IBD. These findings highlight the importance of individualized selection and periodic reassessment of acid suppression therapy as part of personalized management strategies in IBD.

## 1. Introduction

Proton pump inhibitors (PPIs) are among the most frequently prescribed medications worldwide and are commonly used in patients with inflammatory bowel disease (IBD) for gastroesophageal reflux disease, dyspepsia, stress-ulcer prophylaxis, and medication-related upper gastrointestinal symptoms [1,2]. Consequently, a substantial proportion of patients with IBD are exposed to acid-suppressive therapy during their disease. Despite their widespread use, increasing attention has focused on the potential impact of PPIs on intestinal inflammation, infection risk, and IBD course [3,4,5].

PPIs produce profound and sustained gastric acid suppression through covalent inhibition of the H^+^/K^+^-ATPase, resulting in prolonged suppression of gastric acid until new proton pumps are synthesized [6]. Previous studies have demonstrated that PPI use is associated with significant changes in gut microbiota composition, reduced microbial diversity, and impaired colonization resistance [7]. These microbiome effects have been linked to increased susceptibility to enteric infections, particularly *Clostridioides difficile* (*C. difficile*), a complication of particular concern in patients with IBD who frequently receive antibiotics and immunosuppressive therapies [4,8,9]. Given the central role of microbial dysbiosis and immune–microbial interactions in IBD pathogenesis [10,11], these biologic effects raise concern that PPI exposure may influence disease activity and downstream treatment needs.

Observational studies examining the relationship between PPIs and IBD outcomes have yielded inconsistent results. Several population-based analyses and cohort studies have reported associations between PPI use and increased risk of incident IBD or worse clinical outcomes, whereas others have suggested attenuation of these associations after accounting for latency and protopathic bias [3,5,12]. Prior studies have varied in their choice of comparator groups and in how exposure timing and duration were defined, which may influence observed associations. Finally, many studies have focused on incident disease or infection risk, with fewer evaluating downstream clinical outcomes such as steroid exposure or escalation to biologic therapy, which may more directly reflect disease activity and treatment burden in patients with established IBD.

Histamine-2 receptor antagonists (H2RAs), another class of acid-suppressive therapy, provide a clinically relevant active comparator, as they are prescribed for similar indications but exert less profound acid suppression and have more modest effects on the gut microbiome [13]. Comparing PPIs with H2RAs may therefore help disentangle the effects of acid suppression from underlying disease severity and healthcare utilization patterns. In addition, dose–response analyses and a prespecified lag-time (landmark) analysis at 3 months were used to mitigate time-related bias and reduce the influence of early symptom-driven prescribing.

Accordingly, we conducted a large, propensity score-matched cohort study comparing patients with IBD treated with PPIs versus H2RAs. We examined the association between PPI use and subsequent steroid exposure, biologic therapy initiation, *C. difficile* infection, and emergency room (ER)/inpatient visit across multiple follow-up windows. In the context of personalized medicine, understanding whether commonly prescribed acid-suppressive therapies are associated with differential risks of treatment escalation or infection is directly relevant to individualized treatment decisions in IBD.

## 2. Methods and Materials

### 2.1. Data Source

This retrospective cohort study was conducted using the TriNetX [14] analytics platform (TriNetX, 100 Cambridge Park Drive, Suite 501, Cambridge, MA 02140, USA), a de-identified multicenter, global electronic health record (EHR) database from 172 healthcare organizations (HCOs) across 19 countries, comprising more than 169 million patients. TriNetX enables investigators to design cohort queries, perform propensity-score matching, and compare longitudinal outcomes while ensuring compliance with the Health Insurance Portability and Accountability Act (HIPAA) Privacy Rule (§164.514(a)). To protect patient confidentiality, events involving 10 or fewer patients are suppressed and reported as “≤10”. Because this study used only aggregated, de-identified data, it was exempt from Institutional Review Board (IRB) review. This study adheres to the Strengthening the Reporting of Observational Studies in Epidemiology (STROBE) guideline for cohort studies [15] (Appendix A Table A1).

### 2.2. Study Population, Patient Selection, and Exclusions

**Figure 1:**
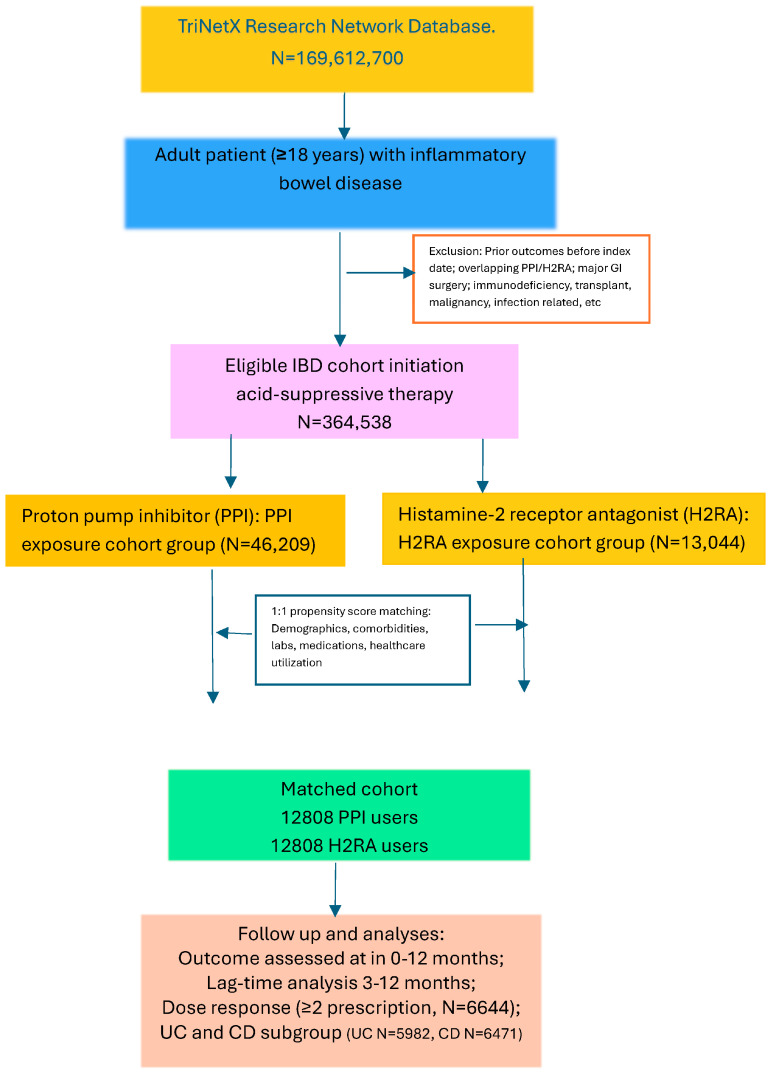
Adult patients with inflammatory bowel disease (IBD) initiating proton pump inhibitors (PPIs) or histamine-2 receptor antagonists (H2RAs) were identified from the TriNetX research network. After 1:1 propensity score matching, outcomes were assessed during 0–12 months with prespecified lag-time, dose-response, and subgroup analyses. (UC: ulcerative colitis; CD: Crohn’s disease).

We identified all non-deceased patients with active records on the TriNetX platform (1 January 2000–1 March 2025) aged ≥18 years (Figure 1). Patients were required to have a documented diagnosis of IBD, including ulcerative colitis (UC, ICD-10 code K51) or Crohn’s disease (CD, ICD-10 code K50), and subsequent exposure to either PPI (omeprazole, pantoprazole, esomeprazole, lansoprazole, or rabeprazole) or H2RAs (cimetidine, famotidine, nizatidine, or ranitidine) during the study period. Query search criteria were based on standard diagnostic, procedural, or medication codes (Appendix A Table A2).

Patients were excluded if they met any of the following criteria: (1) age <18 years at cohort entry; (2) concomitant use of both PPIs and H2RAs at or before the index date; (3) prior major gastrointestinal surgery, including colectomy or other colon surgery, or receipt of colon-related endoscopic procedures before cohort entry; (4) conditions likely to confound immune status or gastrointestinal outcomes, including immunodeficiency disorders, stem cell transplant, organ and tissue transplant, HIV infection, receipt of antineoplastic agents, celiac disease, rheumatoid disease, diverticular disease, intestinal vascular disease, or infectious gastroenteritis; (5) a documented history of the study outcomes prior to the index date, to ensure assessment of incident events. These exclusion criteria were applied uniformly across all analytic cohorts (Appendix A Table A2).

### 2.3. Cohort Construction

For the cohort and study design, see Figure 1. Patients were classified into different exposure cohorts based on medication use at the index date.

(1) Primary-exposure cohort: Patients were classified into (i) a PPI cohort, defined by initiation of any PPI after the first IBD encounter, and (ii) an H2RA cohort, defined by initiation of any H2RA after the first IBD encounter. (2) Dose–response cohort (≥2 prescriptions): To evaluate the impact of sustained exposure, analyses were repeated among patients receiving two or more than two prescriptions of the index medication. (3) Subgroup analysis: Prespecified subgroup analyses were conducted according to IBD subtype (UC or CD). (4) The index date was defined as the first prescription of the index acid-suppressive therapy occurring after the first recorded IBD encounter (The first recorded diagnostic code for UC or CD in the electronic health record). To minimize exposure overlap, patients in the PPI cohort were required to have no H2RA prescriptions after the first IBD encounter, and patients in the H2RA cohort were required to have no PPI prescriptions after the first IBD encounter.

### 2.4. Outcomes

The primary outcomes of interest were (1) systemic steroid exposure, defined as initiation of oral prednisone (>30 mg daily), intravenous (IV) methylprednisolone, oral or IV hydrocortisone, oral budesonide; (2) initiation of biologic therapy, defined as initiation of monoclonal antibody therapies approved for moderate to severe IBD, including anti TNF agents (infliximab, adalimumab, certolizumab, and golimumab); anti-IL-12/23 or anti-IL-23 agents (ustekinumab and risankizumab); anti-integrin therapy (vedolizumab); (3) *C. difficile* infection; and (4) ER/Inpatient encounter. Outcomes were identified using diagnosis and prescription codes recorded in the EHR (Appendix A Table A2). Outcomes were assessed during two prespecified follow-up windows: 0–12 months following the index date, and 3–12 months.

### 2.5. Covariate Selection

Baseline covariates were selected based on clinical relevance and the prior literature. These included demographic characteristics (age, sex, race, and ethnicity); cardiometabolic conditions (hypertension, hyperlipidemia, atherosclerotic cardiovascular disease, heart failure, and peripheral vascular disease); chronic kidney disease; type 1 or 2 diabetes; obesity; gastro-esophageal reflux disease; peptic ulcer disease with/without hemorrhage; medication use (including aspirin, anticoagulants, and high-risk antibiotics); laboratory markers (erythrocyte sedimentation rate, C-reactive protein, and fecal calprotectin); alcohol-related disorder and nicotine-dependent; steroid and immunosuppressant use; and inpatient or ER encounters (all covariates are reported in full in the baseline characteristic Table 1) (diagnostic and prescription codes are listed in Appendix A Table A2).

### 2.6. Primary and Sensitivity Statistical Analysis

Primary analyses: To compare 0–12 months’ outcomes between PPI and H2RA cohorts. Analyses were conducted in the matched cohorts. Follow-up time was calculated from the index date until outcome occurrence, loss to follow-up, or the end of the observation period. To minimize time-related bias, all outcome events occurring prior to the index date were excluded to ensure assessment of incident events.

Sensitivity analyses: Prespecified lag-time analyses were performed, evaluating outcomes during 3–12 months of follow-up, thereby reducing the influence of early symptom-driven prescribing and reverse causation. Dose–response analyses restricted to patients receiving ≥2 prescriptions of the index medication were also conducted. Finally, prespecified subgroup analyses were performed according to IBD subtype, including ulcerative colitis (UC) and Crohn’s disease (CD).

### 2.7. Statistical Analysis

All statistical analyses were conducted on the TriNetX platform or R. Continuous variables are reported as means with standard deviations (SD) and categorical variables as counts and proportions as appropriate. Propensity scores were estimated using logistic regression incorporating all prespecified covariates. Patients were matched 1:1 using nearest-neighbor matching with a fixed caliper. Covariate balance before and after matching was assessed using standardized mean differences (SMDs), with values < 0.1 indicating adequate balance [16,17]. Time-to-event analyses were performed on propensity-matched groups using Kaplan–Meier methods implemented on the TriNetX platform, consistent with the prior literature [18]. Patients were followed up with until the outcome was reached, or were censored after their last data point on record, or when deceased. Log rank testing was performed on the TriNetX platform to assess statistical differences in time to event for each cohort. Hazard ratios (HRs) were estimated using univariate Cox proportional hazards models implemented on the TriNetX platform. TriNetX validated the use of this model with a scaled Schoenfeld residual, wherein a non-random association between these and time is evidence of a violation of the proportional hazard assumption [19]. Additionally, logistic regression models carried out analyses to estimate odds ratios (ORs), which represent the relative odds of experiencing an outcome during the specified follow-up period. Statistical significance was inferred when the 95% CI did not cross unity, corresponding to a two-sided *p*-value < 0.05. Figures were generated in Microsoft Excel and R Studio V.2023.3.0.386 (Boston, MA, USA)

## 3. Results

### 3.1. Baseline Characteristics

A total of 169,612,700 patients were identified within the TriNetX research network. After applying inclusion and exclusion criteria, 364,537 patients with IBD were eligible for analysis. Among these, 46,209 patients initiated PPIs and 13,044 initiated H2RAs. After 1:1 propensity score matching, 12,808 patients were included in each exposure group. After the match, the mean age of the cohort was 48.0 ± 19.0 years; 54% were female, and 45% were male. Baseline demographic characteristics, comorbidities, markers of inflammatory activity, and medications were well balanced between the PPI and H2RA cohorts, with standardized mean differences (SMD) < 0.1 for all variables (Table 1).

### 3.2. Association of PPI Use with Clinical Outcomes

**Figure 2:**
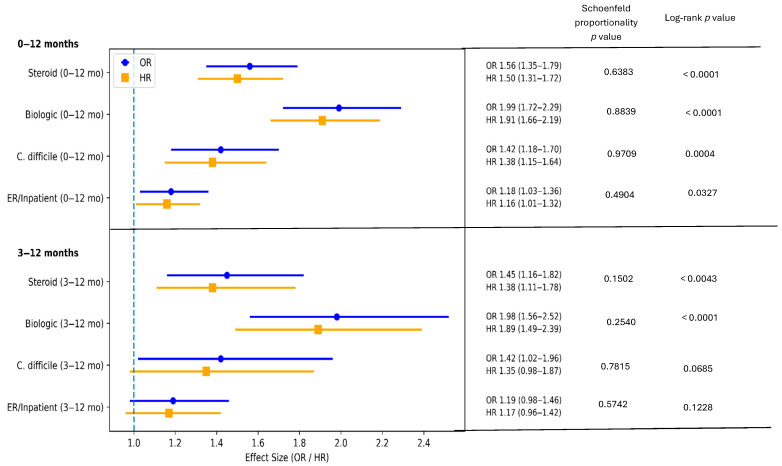
Odds ratios (ORs) and hazard ratios (HRs) comparing PPI versus H2RA use for study outcomes. Error bars represent 95% confidence intervals; the dashed line indicates the null value (OR/HR = 1.0). Time-to-event differences were assessed using log-rank tests; *p* < 0.05 was considered statistically significant. Proportional hazards assumptions were evaluated using Schoenfeld residuals.

**Figure 3:**
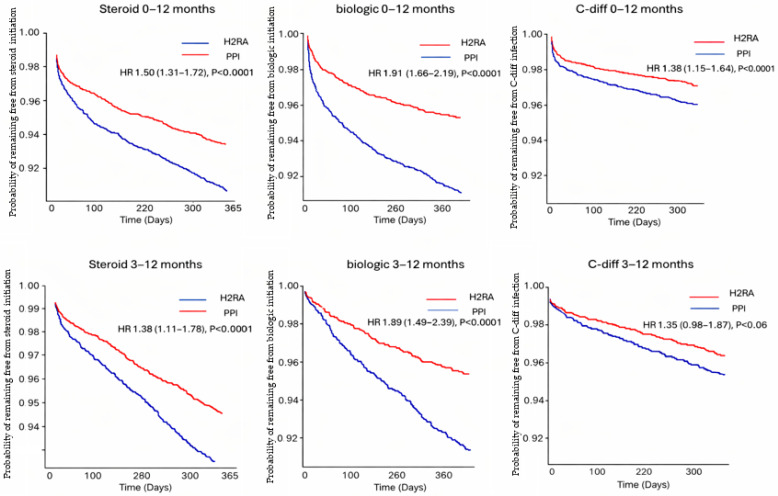
Kaplan–Meier curves comparing time to steroid exposure, biologic therapy initiation, and *Clostridioides difficile* (*C. difficile*) infection between PPI and H2RA cohorts during 0–12 months and 3–12 months of follow-up.

During 0–12 months of follow-up, PPI use was associated with higher ORs and HRs of steroid exposure (OR 1.56, 95% CI 1.35–1.79; HR 1.50, 95% CI 1.31–1.72), initiation of biologic therapy (OR 1.99, 95% CI 1.72–2.29; HR 1.91, 95% CI 1.66–2.19), and *C. difficile* infection (OR 1.42, 95% CI 1.18–1.70; HR 1.38, 95% CI 1.15–1.64). PPI use was also associated with a higher risk of ER/inpatient visits during this period (OR 1.18, 95% CI 1.03–1.36; HR 1.16, 95% CI 1.01–1.32) (Figure 2). Kaplan–Meier analyses demonstrated higher cumulative incidences of systemic corticosteroid exposure, biologic therapy initiation, and *C. difficile* infection among PPI users compared with H2RAs users, with statistically significant early separation and sustained divergence of the curves (Figure 3).

Similar associations were observed during the 3–12-month follow-up window, with persistently increased risks for steroid exposure and biologic therapy initiation. In contrast, the associations with *C. difficile* infection and ER/inpatient visits were attenuated and did not reach statistical significance in time-to-event analyses (Figure 2). The Kaplan–Meier curves are shown in Figure 3.

Also, crude absolute event proportions were calculated among patients at risk during follow-up. During 0–12 months, systemic steroid initiation occurred in 5.50% of PPI users compared with 3.60% of H2RA users (absolute risk difference 1.90%). Biologic therapy initiation occurred in 5.30% versus 2.74%, respectively (absolute risk difference 2.56%). In the 3–12-month lag-time window, biologic initiation occurred 1.90% of PPI users versus 0.97% of H2RA users (absolute risk difference 0.93%), and systemic steroid exposure occurred in 2.02% versus 1.40% (absolute risk difference biologic initiation occurred 1.90% PPI users versus 0.97% H2RA users 0.62%).

### 3.3. Dose–Response Analysis (≥2 Prescriptions)

In analyses restricted to patients receiving ≥ 2 prescriptions, 6644 patients were matched in each group. PPI use for 0–12 months was not significantly associated with steroid exposure or ER/inpatient visits. However, the risk of biologic therapy initiation remained increased (OR 1.56, 95% CI 1.31–1.86; HR 1.54, 95% CI 1.30–1.82). The association with *C. difficile* infection was modest but statistically significant in both odds-based and time-to-event models (OR 1.26, 95% CI 1.02–1.57; HR 1.26, 95% CI 1.02–1.56) (Table 2).

During the 3–12-month period, point estimates for *C. difficile* infection, steroid use, and ER/inpatient visits remained above unity but were not statistically significant. In contrast, the association with biologic (OR 1.48, 95% CI 1.12–1.97; HR 1.45, 95% CI 1.09–1.93) therapy initiation persisted significantly (Table 2).

### 3.4. Subgroup Analysis by IBD Subtype

**Figure 4:**
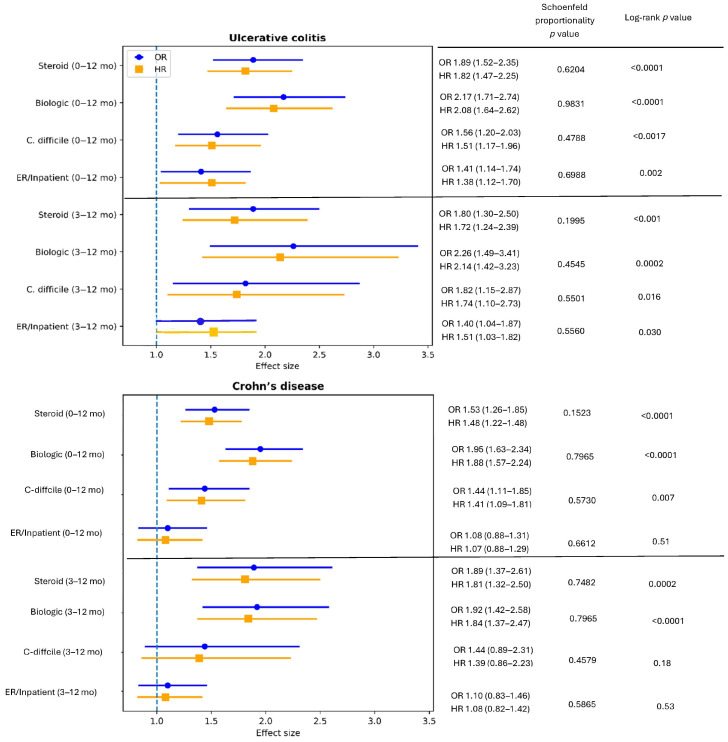
Odds ratios (ORs) and hazard ratios (HRs) for PPI versus H2RA use in ulcerative colitis and Crohn’s disease. Error bars indicate 95% confidence intervals; the dashed line denotes the null value (OR/HR = 1.0). Time-to-event differences were assessed using log-rank tests; *p*-values < 0.05 were considered statistically significant. Proportional hazards assumptions were evaluated using Schoenfeld residuals.

In UC, 5982 patients were matched in each of the PPI and H2RA groups. PPI use was associated with significantly higher risks of steroid exposure, biologic therapy initiation, *C. difficile* infection, and ER/inpatient visits during both the 0–12-month and 3–12-month follow-up windows (Figure 4).

In CD, 6471 patients were matched in each exposure group. During 0–12 months of follow-up, PPI use was associated with increased risks of steroid exposure, biologic therapy initiation, and *C. difficile* infection, whereas no significant association was observed for ER/inpatient visits. During the 3–12-month period, associations with steroid exposure and biologic therapy initiation persisted, while associations with *C. difficile* infection and ER/inpatient visits were attenuated and did not reach statistical significance (Figure 4).

## 4. Discussion

To our knowledge, this is among the first large, real-world, active-comparator studies to examine the association between PPI use and downstream treatment escalation outcomes, *C. difficile* infection in patients with established IBD. We found that PPI use was associated with higher risks of treatment escalation and *C. difficile* infection compared with H2RA use. Across multiple analytic approaches, the most consistent associations were observed for systemic steroid exposure and initiation of biologic therapy outcomes that reflect clinically meaningful disease activity and treatment burden in IBD. These findings extend prior observational work by incorporating active comparator design, incident-event assessment, prespecified follow-up windows, and dose–response analyses to address key sources of bias.

Associations between PPI use and adverse clinical outcomes were strongest during early follow-up (0–12 months). Similar associations were observed during the 3–12-month follow-up window, with persistently increased risks for steroid exposure and biologic therapy initiation. The use of a 3–12 month lag-time window was specifically intended to reduce the influence of early symptom-driven prescribing and reverse causation, which are well-recognized sources of protopathic bias in pharmacoepidemiologic studies [20,21]. However, PPIs are often initiated during periods of symptom exacerbation or early flare-related care. Even with the lag-time analyses, these findings should be interpreted cautiously, and the observed association may partially reflect underlying disease severity rather than direct pharmacologic effect. In contrast, the associations with *C. difficile* infection and ER/inpatient visits were attenuated in the 3–12 months window. Healthcare utilization outcomes also demonstrated greater heterogeneity across analyses and variability by IBD subtype, which is not unexpected given that such outcomes reflect not only disease activity but also symptom perception, access to care, and health-seeking behavior. In addition, ER/inpatient encounters in TriNetX represent all-cause utilization and cannot be specifically attributed to IBD-related activity, which may also contribute to greater heterogeneity in these outcomes and represent an inherent limitation of EHR-based analyses.

Dose-response analyses of patients receiving two or more than two prescriptions further contextualized these findings. Attenuation of some associations in this subgroup likely reflects exclusion of short-term or situational PPI use initiated during periods of acute illness or disease flare, thereby reducing the influence of early symptom-driving prescribing and protopathic bias. In this more stable exposure group, biologic therapy initiation, an indicator of sustained disease severity, remained consistently associated with PPI use. The persistence of associations in the ≥2-prescription analyses raises the possibility of a more sustained association beyond early symptom-driven prescribing. However, residual confounding by underlying disease trajectory cannot be excluded, and these findings should be interpreted cautiously.

Subgroup analyses further demonstrated phenotype-specific patterns. Among patients with UC, PPI use was associated with higher risks of treatment escalation, *C. difficile* infection, and healthcare utilization across both 0–12-month and 3–12-month follow-up periods. In contrast, among patients with CD, associations with steroid exposure and biologic initiation persisted, whereas associations with *C. difficile* infection and ER/inpatient visits were attenuated over time. These differences may reflect underlying phenotypic and pathophysiological distinctions between UC and CD, including differences in disease distribution, mucosal involvement, microbial ecology, and clinical management strategies [22,23]. UC, characterized by continuous colonic involvement, may be particularly susceptible to perturbations in colonic microbiota and mucosal immune responses associated with acid suppression. However, confidence intervals overlapped across subgroups, and formal interaction testing was not performed; therefore, these findings should be interpreted cautiously and not as definitive evidence of differential effects between ulcerative colitis and Crohn’s disease.

Several biologic mechanisms may plausibly link PPI exposure to adverse outcomes in IBD. PPIs induce profound gastric acid suppression, leading to alterations in gut microbiota composition, reduced microbial diversity, and impaired colonization resistance [7,24,25]. Recent meta-analysis indicates an increase in the risk of *C. difficile* infection with increased dose and duration of PPI [26]. In patients with IBD who already exhibit baseline dysbiosis [27,28], impaired mucosal barrier function, and frequent exposure to antibiotics and immunosuppressive therapies, these microbiome perturbations may be particularly consequential, consistent with our observation of increased *C. difficile* infection risk in both UC and CD.

Beyond infection risk, multiple pathways may link PPI exposure to treatment escalation. Dysbiosis and *C. difficile* infection can precipitate mucosal inflammation and disease flares, thereby increasing the need for systemic corticosteroids and subsequent biologic therapy. In addition, PPIs have been shown to impair innate immune function, including neutrophil activity and lysosomal acidification [29], and to alter antigen processing and T-cell activation. Acid suppression has also been associated with increased intestinal permeability [30], potentially facilitating luminal antigen translocation and amplifying mucosal immune responses. Together, these mechanisms may lower the threshold for clinically apparent disease activity and accelerate escalation to immunosuppressive therapies.

This study has several strengths, including a large, well-characterized IBD cohort; use of an active comparator to minimize confounding by indication; comprehensive adjustment for demographic, medication, and laboratory covariates; and multiple prespecified sensitivity analyses addressing dose, timing, and protopathic bias. Nonetheless, limitations should be acknowledged. Metabolic comorbidities and broader patient health factors may influence inflammatory bowel disease progression and treatment decisions [31]. Although several comorbid conditions were included in the propensity matching process, unmeasured or incompletely captured comorbidities may still contribute to residual confounding in real-world observational analyses. Inflammatory biomarkers were incompletely captured, and PPIs initiation may have occurred during early flare-related care. Therefore, observed associations should be interpreted as reflecting treatment patterns within real-world clinical contexts rather than direct causal effects. In addition, treatment escalation endpoints may be associated with provider behavior, regional practice variation, and healthcare access within the TriNetX network. These outcomes, therefore, represent real-world clinical management patterns rather than direct measures of inflammatory severity. The attenuation observed in lag-time and sustained-exposure analyses further supports cautious interpretation of the findings and suggests that early prescribing dynamics and residual confounding may contribute to the observed associations. Medication exposure was based on prescription records and may not fully capture adherence or over-the-counter PPI use. Outcome ascertainment relied on EHR coding, and detailed measures of disease activity, endoscopic findings, and indication for PPI use were not available. Utilization-based outcomes may be influenced by non-IBD-related factors, reflecting a limitation of EHR data. As an observational study, these findings demonstrate associations rather than causation, and residual confounding cannot be excluded.

This study addresses a common clinical dilemma: whether the choice of acid-suppressive therapy matters in patients with IBD who frequently require these medications. By comparing PPIs with H2RAs using a real-world active-comparator design, we provide evidence that therapy selection may be associated with differences in downstream treatment escalation. Although relative risks were elevated, absolute differences were modest. In the 3–12-month window, biologic initiation occurred in 1.90% of PPI users versus 0.97% of H2RA users (absolute difference 0.93%), and systemic steroid exposure differed by 0.62%. Even small differences in escalation risk may influence individualized treatment decisions when multiple acid-suppressive options are clinically appropriate, though causality cannot be established.

## 5. Conclusions

In patients with IBD who require acid-suppressive therapy, the choice of agent may be clinically relevant. In this real-world cohort, PPI use was associated with higher risks of treatment escalation and *C. difficile* infection compared with H2RAs use, with the strongest and most consistent associations observed for systemic corticosteroid exposure and biologic therapy initiation. Although some associations attenuated over time, suggesting a contribution of early symptom-driven prescribing, persistent signals for treatment escalation suggest that the findings are not entirely explained by early symptom-driven prescribing; however, residual confounding by underlying disease severity cannot be excluded. When high-potency acid suppression is not clearly indicated, H2RAs may represent a reasonable alternative, particularly in patients at higher risk for disease exacerbation or infection. Together, these results support judicious selection and periodic reassessment of acid-suppressive therapy as part of a personalized management strategy in IBD.

## Figures and Tables

**Table 1:** Baseline patient characteristics of PPI and H2RA before and after propensity score matching.

Characteristic	Before Match			After Match		
	Cohort, No.		SMD	Cohort, No.		SMD
	PPI	H2RA		PPI	H2RA	
Total No.	46,209	13,044		12,808	12,808	
**A. Demographics**						
Age, mean ± SD (years)	52.1 ± 19.3	47.7 ± 19.0	0.234	48.0 ± 19.3	48.0 ± 19.0	0.004
Female, *n* (%)	22,378 (47.6)	7039 (53.8)	0.126	6922 (54.0)	6836 (53.4)	0.005
Male, *n* (%)	24,591 (52.3)	6037 (46.1)	0.126	5873 (45.9)	5966 (46.6)	0.003
White race, *n* (%)	30,162 (64.2)	9479 (72.5)	0.179	9253 (72.2)	9291 (72.5)	0.001
Not Hispanic or Latino, *n* (%)	26,500 (56.4)	9098 (69.4)	0.2724	8858 (69.1)	8849 (69.0)	0.0015
Black or African American, *n* (%)	2927 (6.2)	1385 (10.6)	0.158	1330 (10.4)	1312 (10.2)	0.015
Hispanic or Latino, *n* (%)	1589 (3.4)	567 (4.3)	0.050	567 (4.4)	549 (4.3)	0.005
Asian, *n* (%)	1149 (2.5)	395 (3.0)	0.0349	383 (3.0)	385 (3.0)	0.0001
Unknown Ethnicity, *n* (%)	18,901 (40.2)	3438 (26.2)	0.3002	3408 (26.6)	3421 (26.7)	0.002
Unknown race, *n* (%)	10,968 (23.3)	1292 (9.9)	0.3684	1349 (10.5)	1292 (10.1)	0.014
American Indian or Alaska Native, *n* (%)	131 (0.3)	44 (0.3)	0.0103	31 (0.2)	44 (0.3)	0.018
Native Hawaiian or Other Pacific Islander, *n* (%)	77 (0.2)	31 (0.2)	0.013	29 (0.2)	30 (0.2)	0.007
**B. Comorbidities**						
Heart failure, *n* (%)	3141 (6.68%)	616 (4.70%)	0.086	598 (4.66%)	616 (4.80%)	0.007
Chronic ischemic heart disease, *n* (%)	5250 (11.17%)	1114 (8.50%)	0.090	1110 (8.66%)	1111 (8.66%)	0.000
Alcohol related disorders, *n* (%)	1852 (3.94%)	424 (3.24%)	0.038	438 (3.42%)	421 (3.28%)	0.007
Overweight and obesity, *n* (%)	5778 (12.29%)	1686 (12.87%)	0.017	1624 (12.66%)	1642 (12.80%)	0.004
Body mass index [BMI] 40.0–44.9, adult, *n* (%)	597 (1.27%)	203 (1.55%)	0.024	220 (1.72%)	195 (1.52%)	0.015
Body mass index [BMI] 30–39, adult, *n* (%)	2411 (5.13%)	763 (5.82%)	0.030	757 (5.90%)	741 (5.78%)	0.005
Type 1 diabetes mellitus, *n* (%)	508 (1.08%)	140 (1.07%)	0.001	124 (0.97%)	138 (1.08%)	0.011
Long-term (current) use of immunosuppressive biologic, *n* (%)	219 (0.47%)	134 (1.02%)	0.065	114 (0.89%)	113 (0.88%)	0.001
Ulcer of esophagus, *n* (%)	322 (0.69%)	20 (0.15%)	0.083	39 (0.30%)	20 (0.16%)	0.031
Ulcer of esophagus without bleeding, *n* (%)	247 (0.53%)	18 (0.14%)	0.068	30 (0.23%)	18 (0.14%)	0.022
Acute peptic ulcer, without hemorrhage or perforation, *n* (%)	77 (0.16%)	10 (0.08%)	0.025	10 (0.08%)	10 (0.08%)	0.000
Chronic peptic ulcer, without hemorrhage or perforation, *n* (%)	20 (0.04%)	10 (0.08%)	0.014	10 (0.08%)	10 (0.08%)	0.000
Acute peptic ulcer, with hemorrhage, *n* (%)	15 (0.03%)	10 (0.08%)	0.019	10 (0.08%)	10 (0.08%)	0.000
COPD, *n* (%)	3182 (6.77%)	631 (4.82%)	0.084	606 (4.73%)	628 (4.90%)	0.008
Hypertension, *n* (%)	15,678 (33.4)	3608 (27.6)	0.126	3673 (28.7)	3584 (28.0)	0.013
GERD, *n* (%)	15,723 (33.5)	2686 (20.5)	0.294	2774 (21.7)	2684 (21.0)	0.053
Type 2 diabetes, *n* (%)	6513 (13.9)	1336 (10.2)	0.112	1338 (10.5)	1333 (10.4)	0.002
Chronic kidney disease, *n* (%)	3551 (7.6)	815 (6.2)	0.052	828 (6.5)	810 (6.3)	0.004
Nicotine dependence, *n* (%)	2747 (5.9)	886 (6.7)	0.038	822 (6.4)	868 (6.7)	0.014
**C. Laboratory Variables**						
CRP, mean ± SD (mg/L)	32.91 ± 56.28	27.44 ± 49.75	0.103	33.05 ± 56.03	27.60 ± 49.85	0.09
CRP ≤ 10 mg/L, *n* (%)	8541 (18.1)	2329 (17.8)	0.010	2210 (17.2)	2256 (17.5)	0.009
ESR, mean ± SD (mm/hr)	24.10 ± 24.48	23.75 ± 24.39	0.015	25.21 ± 24.85	23.84 ± 24.44	0.056
ESR ≤ 20 mm/hr, *n* (%)	6334 (13.4)	2027 (15.5)	0.057	1951 (15.2)	1949 (15.2)	0.000
Hemoglobin A1c, mean ± SD	6.13 ± 1.40	5.99 ± 1.35	0.100	6.05 ± 1.39	6.00 ± 1.35	0.036
HbA1c ≥ 7%, *n* (%)	2242 (4.8)	534 (4.0)	0.034	506 (4.0)	530 (4.1)	0.010
HbA1c ≥ 8%, *n* (%)	1432 (3.1)	355 (2.7)	0.020	343 (2.7)	351 (2.7)	0.004
HbA1c ≥ 9%, n (%)	919 (2.0)	242 (1.8)	0.008	238 (1.9)	240 (1.9)	0.001
Calprotectin, fecal, *n* (%)	1147 (2.4)	486 (3.7)	0.074	474 (3.7)	458 (3.6)	0.007
**D. Medications**						
Anticoagulants, *n* (%)	15,998 (34.0)	4284 (32.7)	0.029	4260 (33.2)	4224 (33.0)	0.006
Methylprednisolone, *n* (%)	7520 (16.0)	3616 (27.6)	0.284	3397 (26.5)	3387 (26.4)	0.002
Prednisone, *n* (%)	8566 (18.2)	2854 (21.8)	0.089	2769 (21.6)	2771 (21.6)	0.000
Ibuprofen, *n* (%)	5238 (11.2)	2299 (17.5)	0.183	2150 (16.8)	2167 (16.9)	0.004
Fluoroquinolones, *n* (%)	8294 (17.7)	2049 (15.6)	0.054	2008 (15.7)	2020 (15.7)	0.003
Aspirin, *n* (%)	8287 (17.6)	1953 (14.9)	0.074	1862 (14.5)	1936 (15.1)	0.016
Hydrocortisone, *n* (%)	5012 (10.7)	2008 (15.3)	0.139	1852 (14.4)	1859 (14.5)	0.002
Budesonide, *n* (%)	4016 (8.6)	998 (7.6)	0.034	1010 (7.9)	975 (7.6)	0.010
Clindamycin, *n* (%)	2119 (4.5)	835 (6.4)	0.082	822 (6.4)	800 (6.2)	0.007
Azathioprine, *n* (%)	3142 (6.7)	622 (4.8)	0.084	628 (4.9)	617 (4.8)	0.004
Naproxen, *n* (%)	2142 (4.6)	585 (4.5)	0.005	552 (4.3)	569 (4.4)	0.006
Vedolizumab, *n* (%)	597 (1.3)	457 (3.5)	0.146	398 (3.1)	356 (2.8)	0.019
Ustekinumab, *n* (%)	665 (1.4)	329 (2.5)	0.079	331 (2.6)	302 (2.4)	0.015
Cephalosporin 3rd generation, *n* (%)	6844 (14.6)	1622 (12.4)	0.064	1574 (12.3)	1598 (12.5)	0.006
Cephalosporin 4th generation, *n* (%)	908 (1.9)	222 (1.6)	0.018	221 (1.7)	221 (1.7)	0.000
Methotrexate, *n* (%)	0 (0%)	0 (0%)		0 (0%)	0 (0%)	
**E. Health Care Utilization**						
Hospital Inpatient and Observation Services, *n* (%)	8577 (18.3)	2268 (17.3)	0.025	2307 (17.9)	2253 (17.6)	0.011
Pulmonary procedures, *n* (%)	4775 (10.2)	1534 (11.7)	0.050	1290 (10.1)	1516 (11.8)	0.056

Abbreviations: SMD = standardized mean difference, SMD < 0.10 indicates adequate covariate balance. COPD: chronic obstructive pulmonary disease; CRP: C-reactive protein; ESR: erythrocyte sedimentation rate. GERD: gastroesophageal reflux disease. PPI = Proton pump inhibito, H2RA = H2 receptor antagonist.

**Table 2:** Association of ≥2 prescriptions of PPI versus H2RA use with clinical outcomes across exposure definitions and follow-up periods.

Exposure Definition	Follow-Up	Outcome	OR (95% CI)	HR (95% CI)
≥2 prescriptions	0–12 months	Steroid	1.15 (0.98–1.35)	1.14 (0.97–1.33)
		Biologic	1.56 (1.31–1.86)	1.54 (1.30–1.82)
		*C. difficile*	1.26 (1.02–1.57)	1.26 (1.02–1.56)
		ER/Inpatient Visit	1.09 (0.92–1.30)	1.11 (0.94–1.31)
	3–12 months	Steroid	1.20 (0.87–1.46)	1.19 (0.93–1.52)
		Biologic	1.48 (1.12–1.97)	1.45 (1.09–1.93)
		*C. difficile*	1.42 (0.95–2.11)	1.40 (0.94–2.09)
		ER/Inpatient Visit	1.12 (0.87–1.45)	1.14 (0.89–1.46)

Odds ratios (ORs) were estimated using logistic regression and hazard ratios (HRs) using Cox proportional hazards models. Confidence intervals are 95%.

## Data Availability

All raw output data from TriNetX are available on reasonable requests by contacting the corresponding author.

## Abbreviations

The following abbreviations are used in this manuscript

IBD	Inflammatory bowel disease
PPI	Proton pump inhibitor
H2RA	H2-histamine receptor antagonist
ER	Emergency room
UC	Ulcerative colitis
CD	Crohn’s disease
*C. difficile*	*Clostridium difficile*

## Appendix A

**Table A1:** STROBE statement—checklist for cohort studies.

Item No.	STROBE Recommendation	Location in Manuscript
1	Identify study design in title/abstract; provide balanced summary	Title; Abstract
2	Explain scientific background and rationale	Introduction
3	State specific objectives and hypotheses	Introduction
4	Present key elements of study design early	Methods: Study Design
5	Describe setting, locations, and relevant dates	Methods: Setting & Data Source
6a	Eligibility criteria, participant selection	Methods: Participants
6b	Matching criteria and numbers matched	Methods: Propensity Score Matching
7	Define exposures, outcomes, confounders, and effect modifiers	Methods: Variables & Definitions
8	Data sources and assessment methods	Methods: Data Sources
9	Describe efforts to address potential bias	Methods: Bias Mitigation
10	Explain study size/rationale	Methods: Study Size
11	Handling of quantitative variables	Methods: Statistical Analysis
12a	Describe all statistical methods, including confounding control	Methods: Statistical Analysis
12b	Methods for subgroup analyses/interactions	Methods: Secondary Analyses
12c	Explain handling of missing data	Methods: Missing Data
12d	Handling of loss to follow-up	Not applicable (retrospective cohort)
12e	Sensitivity analyses	Methods: Sensitivity Analyses
13a	Report numbers at each study stage	Results: Cohort Flow
13b	Give reasons for non-participation	Not applicable
13c	Flow diagram	Methods: patient selection
14a	Characteristics of study participants	Results: Baseline Characteristics
14b	Number with missing data	Methods & Results
14c	Summarize follow-up time	Not applicable
15	Outcome events or summary measures	Results: Cumulative Risk & Event Data
16a	Provide unadjusted/adjusted estimates with precision	Results: Odd ratios, Hazard ratios
16b	Report category boundaries	Methods: Variable Definitions
16c	Translate estimates into absolute risk	Results: Cumulative Risks
17	Report subgroup/sensitivity analyses	Results: Secondary Analyses
18	Summarize key results	Discussion: Summary of Findings
19	Discuss limitations	Discussion: Limitations
20	Interpret results cautiously	Discussion: Interpretation
21	Discuss generalizability	Discussion: Generalizability
22	Funding sources and role	Funding Statement
23	Ethical approval/IRB exemption	Ethics Statement

**Table A2:** Diagnostics, procedure, and medication codes used for query, propensity matching, or outcomes.

Search Terms	Associated Code
Crohn’s Disease	ICD-10: K50 *
Ulcerative Colitis	ICD-10: K51 *
Gastro-Esophageal Reflux Disease	ICD: K21
Scleroderma	ICD-10: M34
Hypertension	ICD-10: I10
Chronic Ischemic Heart Disease	ICD-10: I25
Chronic Obstructive Pulmonary Disease	ICD-10: J44
Lupus Erythematosus	ICD-10: L93
Sjörgen Syndrome	ICD-10: M35.0
Type 1 Diabetes Mellitus	ICD-10: E10
Type 2 Diabetes Mellitus	ICD-10: E11
Overweight and obesity	ICD-10: E66
Stem Cell Transplant	TNX Curated 1005
Antineoplastic Agents	ATC L01
Transplanted Organ and Tissue	ICD-10: Z94
Short Bowel Syndrome	ICD-10: K90.82
Celiac Disease	ICD-10: K90.0
Diverticular Disease of Intestine	ICD-10: K57 *
Acute Vascular Disorder of Intestine	ICD-10: K55.0
Other Chronic Pancreatitis	ICD-10: K86.1
Malignant Neoplasm of Colon	ICD-10: C18
Colorectal surgeries and procedures	CPT: 1007463, 1007422, 1007570, 1007695, 1007952, 1007592, 1007596, 1007620, 1007663, 1007671, 1007687, 1007692, 1007591, 1007692, 45385, 45390, 45382, 45388 SNOMED CT: 4558008, 118839001, 118840004, 118841000, ICD-9: 46.2
Colonoscopy	SNOMED: 73761001
Unspecific Noninfective Gastroenteritis and Colitis	ICD-10: K52
Postsurgical Malabsorption	ICD-10: K91
Ehlers–Danlos Syndrome	ICD-10: Q79.6
Intestinal Adhesions with Obstruction	ICD-10: K56.5
Unspecified Diseases of Spinal Cord	ICD-10: G95
Rheumatoid Arthritis	ICD-10: M06
SLE	ICD-10: L93
Multiple Sclerosis	ICD-10: G35
Amyloidosis	ICD-10: E85
Atherosclerosis	ICD-10: I70
Peripheral Vascular Disease	ICD-10: I73.9
Coronary Artery Disease	ICD-10: I25.1
Heart Failure	ICD-10: I50
Chronic Kidney Disease	ICD-10: N18
Tobacco Use Disorder	ICD-10: F17
Alcohol Use Disorder	ICD-10: F10
Other Specified Noninfective Gastroenteritis and Colitis	ICD-10: K52.8
Noninfective Gastroenteritis and Colitis, Unspecified	ICD-10: K52.9
Chronic Obstructive Lung Disease	ICD-10: J44
Other Unspecified Intestinal Obstruction	ICD-10: K56.6
Fistula of Intestine	ICD-10: K63.2
Intestinal Failure	ICD-10: K90.83
Erythrocyte Sedimentation Rate	TNX Curated 9066
C-reactive protein	TNX Curated 9063
Hemoglobin A1C	TNX Curated 9037
Calprotectin, Fecal	CPT: 83993
Long-Term Use of Immunosuppressive Biologic	ICD-10:Z79.620
Acute Peptic Ulcer without Hemorrhage or Perforation	ICD-10: K27.3
Chronic Peptic Ulcer without Hemorrhage or Perforation	ICD-10: K27.7
Peptic Ulcer with Hemorrhage	ICD-10: K27.0
Ulcer of Esophagus	ICD-10: K22.1
Ulcer of Esophagus without Bleeding	ICD-10: K22.10
Glucagon-like Protein-1 Receptor Agonists	ATC: A10BJ
Proton Pump Inhibitors (PPIs)	RxNorm: 7646, 40790, 283742, 6387, 114979, 1929516
H2-Receptor Antagonist (H2RA)	RxNorm: 4278, 2546, 6056, 9143
Non-Steroid Anti-Inflammatories	RxNorm: 5640, 1191, 7258, 3355, 35827, 41493
Methotrexate	RxNorm:6851
Steroid	RxNorm: 8640, 6902, 5492, 19831
Azathioprine	RxNorm: 1256
Biologic medicine	RxNorm: 1538097, 847083
Human Immunodeficiency Virus (HIV) Disease	ICD-10: B20
Aspirin	RxNorm: 1191
Anticoagulants	VA -: BL 110
Antibiotics	RxNorm: 2582, AM117, AM 118, S01 AE
Hospital inpatient or Observation Service	CPT: 1013659

* Denotes codes that include all subcodes except codes that are denoted by ’. ICD-10 = International Classification of Diseases, Version 10; CPT = Current Procedural Terminology; ATC = Anatomical Therapeutic Chemical Classification System; TNX = TriNetX Curated Codes; and RxNorm = US Normalized Drug Names; SNOMED CT = Systematized Nomenclature of Medicine—Clinical Terms.
